# Factors Associated with Veterinary Professionals’ Intent to Intervene in Cases of Animal Maltreatment

**DOI:** 10.3390/ani16172675

**Published:** 2026-08-26

**Authors:** Kaiulani Long, Chelsea M. Spencer, Ronald Orchard, Cassidy Moreau, Kris Otteman

**Affiliations:** 1 Department of Applied Human Sciences, Kansas State University, Manhattan, KS 66506, USA; cspencer@ksu.edu; 2 College of Veterinary Medicine, Kansas State University, Manhattan, KS 66506, USA; orchard@ksu.edu (R.O.);; 3 Carlson College of Veterinary Medicine and Victim to Verdict, Oregon State University, Corvallis, OR 97331, USA

**Keywords:** animal abuse, veterinary professionals, intervention intentions, animal welfare, veterinary education

## Abstract

This study investigates the factors that contribute to veterinary professionals’ intentions to intervene in cases of suspected animal abuse. A survey was distributed to students, faculty, and staff in two Veterinary Medicine programs. Factors assessed in this study include intentions to intervene, confidence to intervene and educate, time in practice, research knowledge, legal knowledge, procedural knowledge, and experiences applying and educating regarding animal abuse. These variables were assessed through bivariate correlations and path analysis. Results indicate that the perceived seriousness of animal abuse and confidence to intervene are significantly associated with intentions to intervene in cases of suspected animal abuse. In addition, perceived seriousness of animal abuse, procedural knowledge, and applied and educational experiences in relation to animal abuse are significantly associated with confidence to intervene and educate in cases of suspected animal abuse. Confidence was found to mediate the effect that procedural knowledge has upon intentions to intervene. Veterinary professionals’ intentions to intervene in cases of suspected animal abuse may depend upon factors beyond their knowledge of how to intervene; such factors may include the perceived seriousness of such abuse, their procedural knowledge, and their confidence in their abilities to intervene.

## Simple Summary

Animal abuse remains an important animal welfare concern, and veterinary professionals are often in a unique position to recognize and respond to suspected cases. Using a survey of veterinary students, faculty, and staff, we found that individuals who perceived animal abuse as more serious and who felt more confident in their ability to respond were more likely to intend to intervene. The findings suggest that increasing intervention may require more than knowledge alone and that training opportunities focused on practical skills and confidence-building may help veterinary professionals feel better prepared to respond to suspected animal maltreatment.

## 1. Factors Associated with Veterinary Professionals’ Intent to Intervene in Cases of Animal Maltreatment

Animal abuse is a serious issue that warrants significant attention. Clinical and animal welfare frameworks define animal cruelty as acts of neglect, deprivation, or intentional harm that compromise an animal’s well-being, and such harm can occur through either omission (e.g., denial of care) or commission (e.g., direct abuse) [1,2,3]. Identifying prevalence rates of animal abuse is difficult due to issues of underreporting and inconsistency in how the laws define abuse [4,5,6,7]. However, investigations of reported cases show that common forms of animal abuse include active cruelty, neglect, and unsafe living conditions [8,9]. Additionally, research has identified that animal abuse perpetration is associated with other acts of violence and coercive control, such as intimate partner violence, child abuse, and general antisocial tendencies [10,11,12,13,14,15]. Continued examination of ways to protect animals and intervene in cases of animal abuse is needed.

## 2. Veterinarians and Animal Abuse Intervention

Veterinarians are uniquely positioned to identify and document physical findings in cases of animal abuse that would likely go unreported as part of their routine clinical encounters [1]. Veterinary practitioners are able to observe, document, and communicate with caregivers regarding injuries or signs of neglect as they become apparent [8]. Professional organizations have increasingly emphasized the role of veterinary professionals in responding to suspected animal abuse. The AVMA Principles of Veterinary Medical Ethics underscore veterinarians’ responsibilities to protect animal welfare and prevent suffering. Similarly, the AVMA Position Statement on Animal Abuse and Neglect and the AAHA Animal Abuse Reporting Position Statement encourage veterinary professionals to recognize, document, and appropriately respond to suspected abuse and neglect. Together, these guidelines reinforce the important role veterinary professionals play in identifying and addressing animal maltreatment [16,17,18].

Studies have found that even when veterinary practitioners perceive abuse, most indicate confusion in identifying and reporting abuse [1,19,20]. However, ethically, veterinary practitioners should report potential abuse to protect animal welfare [5,21]. Similarly, foundational literature has been warning for decades that practitioners cannot report cruelty without training, and recent guidance continues to emphasize the competencies of recognizing, documenting, and reporting animal abuse [1,22,23].

The degree to which a veterinarian may choose to intervene depends upon preparation, practice, and institutional support [1,19,24]. For example, integrating welfare sciences, ethics, legal frameworks, shelter medicine, partnerships, and forensic training have been shown to enhance professionals’ capacity to recognize, document, and address animal abuse cases [3]. However, similar to other aspects of education, welfare science and related concepts are often not consistently applied or accessible to forensic training [3,25,26]. Studies have also pointed to a lack of available training and deficient workplace policies as two significant obstacles to intervening in cases of animal abuse [5,27].

Research has shown significant relationships between knowledge, perception of seriousness, and confidence in intervening in animal abuse cases [3,28,29,30]. Although knowledge and experience are important predictors of intervention behavior, prior research suggests that multiple organizational, interpersonal, and legal factors may influence veterinary professionals’ willingness to intervene. These barriers may include uncertainty regarding workplace policies and reporting procedures, concerns about confidentiality, time and economic constraints, fear of damaging veterinarian-client relationships, concerns that reporting may not result in meaningful action, and uncertainty about which agencies should receive reports [1,5]. These findings suggest that confidence may be influenced by broader professional and contextual factors beyond knowledge alone [6]. Professional competence can encompass the amount of time spent practicing, the behavioral abilities developed through education and practice, legal knowledge, and procedural knowledge gained through education; and perceived seriousness is defined as the degree to which professionals perceive an obligation to intervene in response to abuse [6,26,28]. Further examination of factors associated with professional competencies and their associations with veterinarians’ intentions to intervene in cases of animal abuse is needed.

Additionally, veterinary professionals require both self-efficacy and confidence in intervening in suspected cases of animal abuse, as many veterinarians who suspect animal abuse will fail to report due to lack of procedural certainty [10,19,20]. Ferrell identified significant variations in the inclusion of veterinary forensic science courses at U.S. veterinary schools [3]. Education in Veterinary Forensic Science has been demonstrated to improve students’ competencies to identify patterns of injury, gather and preserve evidence, maintain accurate records, and distinguish between accidental and non-accidental injuries [28]. Students experience increased confidence to act upon situations where abuse may exist and develop an ability to express concerns about potential animal cruelty and take the appropriate steps to report such incidents [28]. Further exploration of how confidence is associated with veterinarians’ intentions to intervene in cases of animal maltreatment or neglect is warranted.

Veterinary professionals do not represent a homogeneous workforce. Individuals working in academic veterinary medicine, private practice, shelter medicine, corporate practice, and large- and small-animal settings may differ substantially in their exposure to suspected animal cruelty, workplace expectations, reporting responsibilities, and available institutional supports [3,5]. These differences may influence both confidence and willingness to intervene in suspected abuse cases [6]. Therefore, understanding factors associated with intervention intentions across different segments of the veterinary community remains an important area of research.

Although prior research has identified relationships among knowledge, perceived seriousness, and confidence, less is known about how these factors function together to predict veterinarians’ intentions to intervene, particularly within a unified theoretical framework. Therefore, the purpose of this study is to examine how professional competencies, including knowledge (research and legal), experience (years in practice, number of educational and training experiences), and perceived seriousness, relate to veterinarians’ intentions to intervene, with confidence conceptualized as a potential mediating mechanism.

## 3. Theoretical Framework

Social Cognitive Theory provides a framework for understanding the relationships among behavior and the presence of knowledge, experiences, perceived consequences, and efficacy beliefs [31]. The approach to animal cruelty is consistent with the decision-making process that veterinarians follow when deciding whether to intervene in cases of animal abuse. The model of animal cruelty defines the three bases of recognizing and responding to animal abuse, including harms, norms, and control. These definitions align with the decision-making process used by veterinarians to intervene in cases involving animal abuse [1]. Predictors in this study include time in practice, educational exposures, knowledge of laws and procedures, and perceived severity. Confidence to intervene in cases of animal abuse was identified as a mediator, which would help convert knowledge into actions. Based on this paradigm, proficiency is defined as the time spent in practice; behavioral capability is defined as the knowledge gained through education and practical application, including knowledge of procedures and laws, while perceived seriousness defines whether the abuse is serious enough to warrant an intervention [6,26,28]. Confidence to intervene and self-efficacy are critical, as studies of violence intervention have shown that those who perceive themselves as capable of applying their skills to violent situations, who have learned the process of intervening in such situations, and who are confident that they will be successful under pressure are significantly more likely to act [32,33]. Within this framework, procedural knowledge and educational experiences may contribute to behavioral capability, perceived seriousness reflects outcome expectations, and confidence represents self-efficacy, which together influence intervention behavior.

## 4. The Present Study

The current study aims to examine factors associated with veterinarians’ intent to intervene in suspected cases of animal abuse, with a focus on how professional competencies translate into action. Previous research has identified gaps in training, inconsistencies in identifying abuse, and barriers to intervention [3,5,6,23,26]. However, less is known about how these factors operate together to influence intervention intentions. Guided by Social Cognitive Theory, the present study examines the relationships among professional characteristics (time in practice, perceived seriousness of animal abuse, research knowledge, legal knowledge, procedural knowledge, as well as applied and educational experiences related to animal abuse), perceived seriousness of animal abuse, confidence to intervene, and intentions to intervene in cases of animal abuse. Specifically, confidence to intervene and educate in cases of animal abuse is conceptualized as a mediating mechanism through which knowledge and experience may influence behavioral intentions. This study aims to clarify the mechanisms through which knowledge and experience translate into action, thereby addressing a critical gap in the literature on veterinary responses to animal abuse.

## 5. Method

### Participants and Procedures

The current study was approved by Kansas State University’s Institutional Review Board. Surveys were sent out to students, faculty, and staff in Veterinary Medicine programs at two universities, one located in the Midwest and the other in the Northwest. Potential participants were sent an email describing the survey with a Qualtrics link to participate. All responses were anonymous. There was no compensation for completing the survey. Two reminder emails were sent regarding the survey. Data were collected between January and July of 2025. A total of 145 individuals completed the survey. Of the 145 participants, 71 were veterinary students (49%), 31 (21.4%) were veterinarians, 16 (11%) were veterinary technicians, 12 (8.3%) reported “other,” 11 (7.6%) were academics, two (1.4%) were veterinary paraprofessionals, and two (1.4%) were animal welfare professionals.

## 6. Measures

*Intent to Intervene.* Participants were asked 9 questions regarding their intentions to intervene in cases of animal cruelty. Questions were asked on a Likert-style scale where 1 = *strongly disagree* and 5 = *strongly agree*. Questions asked about their intent to intervene in various types of animal abuse (e.g., physical abuse, neglect, psychological harm) and species of animal abuse (e.g., cat, dog, small pet). The mean was used in the analysis, where higher scores indicated stronger agreement that they would intervene in cases of animal cruelty. The reliability was acceptable (α = 0.96).

*Confidence to Intervene and Educate.* Participants were asked 8 questions about how confident they would be regarding various areas of intervening or educating others regarding animal abuse. Example questions include “How confident would you feel contributing to an investigation of suspected animal cruelty at the request of law enforcement or the court, for example as an expert witness, lead veterinarian, or other contributor?”, “How confident do you feel in your ability to discern an accidental injury from a non-accidental injury of an animal?,” and “How confident are you in your ability to educate other professionals about the seriousness of animal cruelty?” Questions were asked on a 4-point Likert scale where 1 = *not at all confident* and 4 = *very confident*. Mean scores were used, and higher scores indicated higher levels of confidence in intervening and educating others regarding animal cruelty. Reliability was acceptable (α = 0.86).

*Time in Practice.* Participants were asked how long they have been in practice. They were able to choose between currently in school/not yet graduated, less than one year, 1–2 years, 3–5 years, 6–10 years, 10–15 years, 15–20 years, and 20+ years. Higher scores indicated having practiced veterinary medicine for a longer period of time.

*Perceived Seriousness of Animal Abuse.* In order to measure perceived seriousness of animal abuse, participants were asked how serious they believed four different forms of animal abuse (physical, sexual, psychological, neglect) were across four animal categories (cat, dog, small pet, horse), resulting in 16 items. One additional item asked about perceived seriousness of dog fighting, resulting in a total of 17 questions. Psychological abuse was included because the measure was intended to assess participants’ perceptions of the seriousness of different forms of animal abuse, rather than whether specific behaviors met statutory definitions of animal cruelty. Items were scored on a 5-point Likert scale where 1 = not at all serious and 5 = extremely serious. Scores were averaged, and higher scores indicated higher levels of perceived seriousness of various types of animal cruelty. Reliability was acceptable (α = 0.94).

*Research Knowledge.* To assess knowledge related to research on animal abuse, participants were asked 10 true-or-false questions related to current research on the topic. Sample questions include “cats are killed at significantly higher rates in cases of animal cruelty than are dogs” and “men are more likely to commit animal abuse compared to women.” For each answer correct, a 1 was added to their score. This ended with potential scores ranging between 0 and 10, with higher scores indicating higher levels of knowledge regarding research on animal abuse.

*Legal Knowledge.* We asked participants what state they were currently living in (one of two states data were collected from). For each state, we created 15 true-or-false and multiple-choice questions testing their knowledge of the laws regarding animal abuse in their state. Sample questions include “a person can be charged with cruelty to animals even if they did not intend to harm the animal,” “providing adequate veterinary medical care does not absolve an individual from charges of animal cruelty,” and “When a veterinarian in [state] suspects aggravated animal abuse, which of the following actions are they legally required to take?” For each correct answer, a 1 was added to their score. Scores ranged from 0 to 15, with higher scores indicating higher levels of legal knowledge related to animal abuse in their state.

*Procedural Knowledge.* To examine knowledge of procedures surrounding animal abuse, we asked participants three questions on how much they agreed, on a Likert scale where 1 = strongly disagree and 5 = strongly agree, with the following statements: “I know when to report animal cruelty to law enforcement,” “I know how to find the animal protection laws in my area,” and “I understand all the ways in which I, as a member of the veterinary industry or other animal or enforcement-related profession, can contribute to suspected or ongoing animal abuse cruelty cases.” Scores were averaged, and higher scores indicated higher levels of agreement with knowing procedures related to animal cruelty. The reliability was acceptable (α = 0.71).

*Applied and Educational Experiences Related to Animal Abuse.* Participants were asked to select all of the educational experiences they have had so far related to animal abuse. There were ten options, including a course on animal cruelty or forensics, shelter rotation, experience working on animal cruelty cases, webinars, conference presentations, an undergraduate course, a graduate course, a veterinarian school, a criminal justice program, or “other.” Items were summed, where scores could range from 1 to 10, with higher scores indicating more applied and educational experiences related to animal abuse.

## 7. Analysis Plan

First, bivariate correlations were run using SPSS V. 30 [34]. Next, in order to examine whether confidence to intervene and educate regarding animal abuse mediated the relationships between professional characteristics (e.g., time in practice, perceived seriousness of animal abuse, research knowledge, legal knowledge, procedural knowledge, as well as applied and educational experiences related to animal abuse) and intent to intervene, a path analysis was run using MPlus 8.0 [35]. The professional characteristic variables were specified as predictors of confidence to intervene and educate, which was specified as a predictor of intent to intervene. Direct paths from all predictors to intent to intervene were also included, allowing for the examination of partial mediation. This model allowed for the simultaneous estimation of direct and indirect associations. Model parameters were estimated using the maximum likelihood estimator with robust standard errors (MLR). Since the data were cross-sectional, indirect effects were not interpreted as causal mediation but as statistical associations. The model was just-identified (*df* = 0) and did not provide interpretable model fit.

## 8. Results

Bivariate correlations found that perceived seriousness of animal abuse (*r* = 0.492, *p* < 0.001; see Table 1), the number of applied and educational experiences surrounding animal abuse (*r* = 0.272, *p* = 0.002), procedural knowledge of what to do in cases of animal abuse (*r* = 0.352, *p* < 0.001), and confidence to intervene and educate in cases of animal abuse were significantly correlated with intentions to intervene in cases of animal abuse. Perceived seriousness of animal abuse (*r* = 0.294, *p* = 0.001), number of applied and educational experiences (*r* = 0.399, *p* < 0.001), and procedural knowledge (*r* = 0.580, *p* < 0.001) were significantly related to confidence to intervene and educate. Procedural knowledge was significantly related to the perceived seriousness of animal abuse (*r* = 0.217, *p* = 0.018). Legal knowledge and research knowledge were significantly related (*r* = 0.228, *p* = 0.021). Lastly, research knowledge was negatively associated with the amount of time in practice (*r* = −0.282, *p* = 0.003).

**Figure 1:**
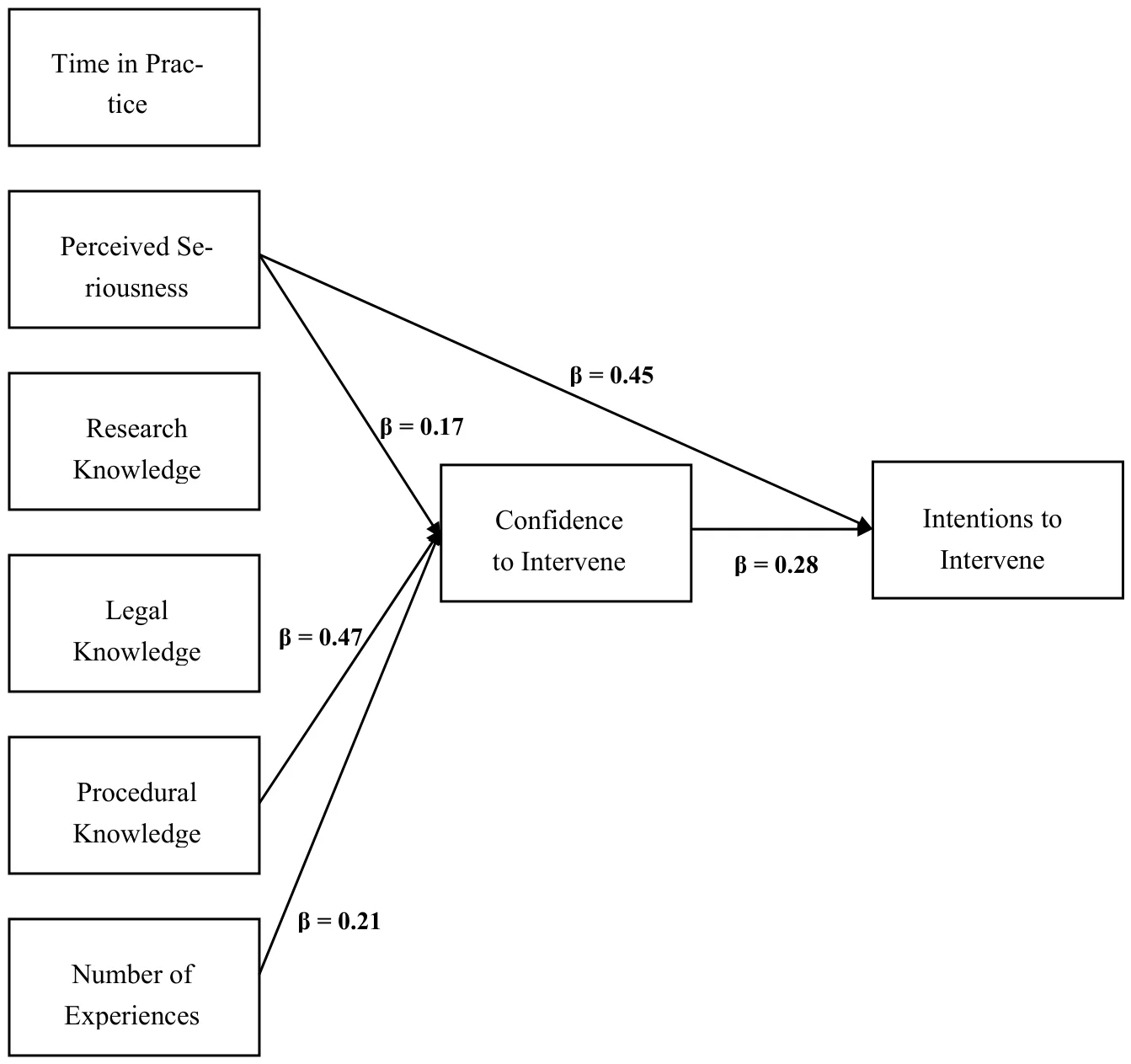
Mediation path analysis examining if confidence to intervene and educate in cases of animal abuse mediates the relationship between professional characteristics and the intent to intervene in cases of animal abuse (*n* = 145).

The path analysis found that confidence in intervening and educating in cases of animal abuse was significantly associated with intentions to intervene in cases of animal abuse (*β* = 0.30, *p* = 0.026; see Table 2 and Figure 1). The perceived seriousness of animal abuse was also significantly related to intentions to intervene in cases of animal abuse (*β* = 0.58, *p* < 0.001). The amount of time in practice, knowledge of research regarding animal abuse, legal knowledge, procedural knowledge of what to do in cases of animal abuse, and the number of educational and applied experiences related to animal abuse were not significantly related to intentions to intervene in cases of animal abuse.

The perceived seriousness of animal abuse (*β* = 0.17, *p* = 0.031), the number of applied and educational experiences related to animal abuse (*β* = 0.21, *p* = 0.006), and procedural knowledge of animal abuse cases (*β* = 0.47, *p* < 0.001) were all significantly associated with confidence in intervening and educating regarding animal abuse. The amount of time in practice, knowledge of research regarding animal abuse, and legal knowledge were not associated with confidence in intervening and educating. One significant indirect path was identified. Confidence to intervene and educate regarding animal abuse mediated the relationship between procedural knowledge of what to do in cases of animal abuse and intentions to intervene (indirect effect = 0.09, *p* = 0.033).

## 9. Discussion

The current study sought to examine factors associated with intention to intervene in cases of animal abuse among students, faculty, and staff in veterinary medicine programs. We found that perceived seriousness of animal abuse, procedural knowledge of what to do in cases of animal abuse, and number of applied and educational experiences related to animal abuse were significantly related to confidence in intervening in cases of animal abuse. We also found that confidence to intervene and perceived seriousness of animal abuse were significantly associated with intentions to intervene in cases of animal maltreatment. There was also a significant indirect path among procedural knowledge related to animal abuse, confidence to intervene, and intentions to intervene. The results from the present study help identify pathways to potentially increase veterinary professionals’ intentions to intervene in cases of animal cruelty.

Perceived seriousness of animal abuse was associated with intentions to intervene in cases of suspected abuse. Perceived seriousness is a factor that considers the reasons why individuals believe that animal abuse should be recognized and acted upon. Perceived seriousness is related to factors like neglect of an animal, deprivation of an animal of the care that it requires, physical abuse, psychological abuse of an animal, and the failure of those who are responsible for an animal to provide the care for that animal [1]. This factor emerged as one of the strongest predictors of intentions to intervene. Perceived seriousness was also associated with confidence to intervene and educate, though the association between these two variables is weaker than the association between procedural knowledge and confidence.

Confidence to intervene and educate in cases of suspected animal abuse was significantly associated with intentions to intervene in those cases. These two variables are correlated with one another in the Social Cognitive Theory model [31]. Confidence was also associated with procedural knowledge, experiences applying the knowledge of how to intervene in cases of animal abuse, experiences educating others regarding animal abuse, and the perceived seriousness of the concept. While training-related factors were associated with confidence in the present study, confidence likely develops through a combination of educational, professional, and organizational influences. Veterinary professionals may possess adequate knowledge of animal cruelty while still feeling uncertain due to workplace culture, unclear reporting procedures, and fear of negative reactions from clients and colleagues. Future research should examine these broader influences on confidence and intervention behavior. The strength of the correlation between confidence to intervene in animal abuse and educational efforts and procedural knowledge suggests that confidence develops in individuals in response to their understanding of the steps that are necessary to intervene in those cases. The confidence that professionals have in their abilities to respond appropriately to animal abuse is also built through educational efforts and training that is provided to veterinary students and professionals regarding the recognition of abuse, the documentation of that abuse, and the reporting of those instances of abuse [28]. Thus, recognizing that confidence was a mediating factor between procedural knowledge and intentions to intervene suggests that educational efforts for veterinary students and professionals may have an impact on their confidence to act in these cases.

The present findings suggest that knowledge alone may be insufficient to predict veterinarians’ intentions to intervene in cases of suspected animal abuse. Current literature suggests that procedural knowledge and applied experience lead to increased confidence and intentions to intervene [3,28]. Knowledge alone may not translate into intervention; however, procedural knowledge appears to be indirectly linked to intervention intentions through confidence, supporting the argument that individuals with higher confidence are more willing to intervene [3,28].

The present findings, in conjunction with prior literature, suggest that ambiguity surrounding accidental versus intentional harm appears associated with hesitancy to intervene [5,6,28]. This uncertainty may contribute to lower confidence, which the current findings suggest plays an important role in individuals’ intentions to intervene. The data also suggest that training opportunities related to identifying and responding to animal cruelty may be limited or inconsistent across veterinary programs, potentially contributing to gaps in procedural knowledge. Without the skills necessary to identify potential animal cruelty and the institutional knowledge of how to appropriately respond when concerns arise, practitioners may feel less confident intervening. Additionally, inconsistencies in state reporting laws and concerns regarding retaliation or lack of legal protections may further contribute to hesitation surrounding intervention and reporting.

The current findings indicate that greater time in practice was negatively associated with current research exposure. This may suggest that experience alone may not always be associated with the growth of confidence in reporting, nor does exposure alone appear sufficient to support intervention intentions. Several explanations may account for this relationship. Practitioners who completed their professional training earlier in their careers may have received less formal education regarding veterinary forensics, animal cruelty reporting, and the links between animal abuse and interpersonal violence than more recent graduates [3,22,23]. Additionally, veterinary professionals in different career stages may face distinct workplace responsibilities and professional pressures that influence their engagement with emerging research and intervention practices. Because these factors were not directly measured in the present study, future research should examine the role of continuing education, workplace responsibilities, practice ownership, and professional culture in shaping intervention intentions.

## 10. Implications

The findings from the present study have several implications for veterinary education, clinical practice, and animal welfare intervention. First, the results suggest that efforts to increase veterinary professionals’ responses to suspected animal abuse should move beyond general knowledge acquisition and prioritize the development of applied confidence. Although research knowledge and legal knowledge were not directly associated with intentions to intervene, procedural knowledge was indirectly associated with intentions through confidence to intervene and educate. This finding suggests that knowing what to do, when to act, and how to navigate reporting or referral processes may be especially important for translating concern about animal abuse into professional action. In practical terms, veterinary curricula and continuing education programs may be strengthened by providing clear, jurisdiction-specific reporting procedures, documentation protocols, and opportunities to rehearse decision-making in suspected abuse cases [36].

Second, perceived seriousness of animal abuse emerged as one of the strongest predictors of intentions to intervene. The perceived seriousness of an animal abuse case may also be influenced by recognition that animal cruelty frequently co-occurs with interpersonal violence, child maltreatment, and elder abuse. Awareness of these links may increase professionals’ perceptions that intervention is necessary and may strengthen support for One Health and One Welfare approaches that recognize the interconnected well-being of humans and animals [14,37]. This finding indicates that veterinary professionals’ appraisal of animal cruelty as a serious welfare concern may be central to whether they intend to take action. Educational programs should therefore not only teach legal definitions or clinical indicators of abuse but should also emphasize the animal welfare consequences of neglect, physical harm, psychological harm, and other forms of maltreatment. Training that helps veterinary professionals recognize the severity of different abuse presentations may strengthen their perceived obligation to intervene, particularly in cases that are ambiguous, chronic, or involve less visible forms of harm. This is especially relevant because animal cruelty can occur through both acts of commission and omission, including direct abuse, deprivation, unsafe living conditions, and denial of needed care [1].

Third, the present findings support the value of experiential and applied learning. The number of applied and educational experiences related to animal abuse was significantly associated with confidence to intervene, even though it was not directly associated with intervention intentions. This pattern suggests that experiences such as animal cruelty or forensic coursework, shelter rotations, webinars, conference presentations, and exposure to cruelty-related cases may help build the self-efficacy needed to respond effectively. For veterinary students and professionals, scenario-based training, case simulations, mock demonstration exercises, interdisciplinary case reviews, and guided practice with reporting pathways may be particularly useful [6,38]. These forms of training may help bridge the gap between recognizing possible cruelty and feeling prepared to act.

Fourth, the results have implications for veterinary workplaces and institutions. Because procedural knowledge was linked to confidence, clinics, hospitals, shelters, and veterinary teaching programs may benefit from developing written protocols for suspected animal abuse. Such protocols could include guidance on recognizing warning signs, documenting clinical findings, preserving evidence, communicating with clients, consulting supervisors or legal authorities, and making reports when appropriate. Institutional policies may also reduce uncertainty among veterinary professionals by clarifying roles and responsibilities before a suspected abuse case arises [1,6]. These supports are important because veterinarians may be uniquely positioned to identify cases of animal abuse that would otherwise remain undetected, but prior research has repeatedly identified uncertainty, lack of training, and unclear workplace procedures as barriers to reporting and intervention [6]. Further understanding about the perception of severity of abuse may help to clarify the educational interventions that have the potential to provide the greatest impact.

It is important to recognize that intervention decisions occur within broader social and community contexts. Veterinary professionals must often balance animal welfare concerns with an understanding of caregiver circumstances and available community resources. Depending on case characteristics, appropriate responses may include reporting to authorities, client education, referrals to supportive services, subsidized veterinary care, or collaboration with animal welfare organizations. Consistent with the One Welfare framework, effective intervention requires consideration of both animal well-being and the broader circumstances affecting caregiving capacity [39]. Therefore, educational efforts may benefit from incorporating ethics, communication skills, interdisciplinary collaboration, and knowledge of available community resources alongside legal and forensic competencies.

This does not diminish the importance of responding to animal suffering. Rather, veterinary training should prepare professionals to assess the severity and context of animal welfare concerns and to respond proportionately. Some situations may require urgent protective action, mandated reporting, or involvement of legal authorities, whereas others may be more effectively addressed through client education, resource referral, subsidized veterinary care, temporary assistance, social-service collaboration, or partnerships with community-based animal welfare organizations. A One Welfare approach requires attention not only to the safety and well-being of animals but also to the social conditions that shape caregiving capacity and the consequences of law enforcement intervention [39]. Thus, cruelty-response training should include legal and forensic competencies alongside ethics, communication, cultural humility, structural competency, veterinary social work, and knowledge of non-carceral or supportive intervention options when appropriate. As veterinary medicine continues to expand its role in identifying and responding to suspected animal cruelty, educational approaches that integrate forensic knowledge with ethical reasoning, structural awareness, and One Welfare principles may better equip professionals to make decisions that protect animals while minimizing unintended harms to people and communities.

## 11. Limitations and Future Research

There are several limitations in the present study that warrant attention. First, the data collected in the present study were cross-sectional. Therefore, we were unable to identify causal mediations or causal pathways to examine ways to improve intentions to intervene in cases of animal abuse. Future research could examine longitudinal data to examine how factors predict intentions to intervene in cases of animal abuse over time. It may also be helpful to examine how specific experiences or interventions impact intentions to intervene using pre- and post-test data. Another limitation of the present study is that data were collected from university samples. Participants were recruited from veterinary medicine programs at two universities and consisted primarily of veterinary students, with fewer practicing veterinarians and veterinary technicians. As a result, the findings may not generalize to the broader veterinary profession. Students and academic personnel may differ from practicing clinicians in their level of exposure to animal cruelty cases, reporting responsibilities, decision-making authority, and workplace constraints. Additionally, not all participants were currently practicing veterinary professionals, which limits conclusions regarding veterinary practitioners specifically. Future research should recruit larger samples of practicing veterinarians and veterinary technicians from a variety of professional settings, including private practice, shelter medicine, corporate veterinary medicine, and academic medicine. Future studies should also examine whether factors associated with intervention differ across large-animal and small-animal practice settings. Additionally, participants were sent an email describing the study and voluntarily took the survey. This may have led to a bias among the sample leaning towards individuals who may be more passionate about the topic of animal abuse. Lastly, the study only collected data from two universities, which may further limit the generalizability of the findings.

## 12. Conclusions

The factors that are associated with veterinary professionals’ intentions to intervene in cases of suspected animal abuse include the seriousness of the abuse and the confidence that they have in their abilities to intervene. Factors that are associated with confidence to intervene and educate include their knowledge of animal abuse, their procedural knowledge to intervene, their experiences applying that knowledge and educating others about the abuse in their careers, as well as the seriousness of animal abuse. However, knowledge of the research related to abuse of animals and knowledge of the laws regarding animal abuse were not associated with intentions to intervene. Such results suggest that education and training in veterinary schools beyond the general knowledge required to recognize animal abuse may be important for increasing their confidence and their intentions to intervene in those cases.

Beyond the education of veterinary students regarding animal abuse, implementing policies within veterinary clinics, shelters, hospitals, and organizations may help to increase knowledge of how to respond to cases of animal abuse. In responding to animal abuse, these professionals must recognize the factors that contribute to the occurrence of abuse. While the response to abuse may include the protection of those animals from further abuse, other responses may include educating those animals’ owners of their responsibilities toward the animals or providing resources and subsidized care for those animals. Finally, while the information that was gathered from these two universities does shed light upon the factors that influence veterinary professionals’ intentions to intervene in cases of suspected abuse, future research in these populations could investigate whether implementing educational experiences into veterinary programs can lead to positive changes in those professionals’ confidence to intervene, their decisions to intervene, and an understanding of the veterinary professionals’ essential role in prevention and response to animal cruelty.

## Figures and Tables

**Table 1:** Correlations among variables used in the analysis (*n* = 145).

	Time in Practice	Perceived Seriousness of Animal Abuse	Research Knowledge	Legal Knowledge	Number of Applied and Educational Experiences	Procedural Knowledge	Confidence to Intervene	Intentions to Intervene
Time in Practice	–							
Perceived Seriousness of Animal Abuse	−0.139	–						
Research Knowledge	−0.282 **	0.043	–					
Legal Knowledge	−0.035	−0.118	0.228 *	–				
Number of Applied and Educational Experiences	−0.047	0.069	0.075	0.139	–			
Procedural Knowledge	−0.134	0.217 *	−0.117	0.154	0.058	–		
Confidence to Intervene	−0.077	0.294 **	−0.035	0.099	0.399 ***	0.580 ***	–	
Intentions to Intervene	0.031	0.492 ***	−0.071	0.004	0.272 **	0.352 ***	0.474 ***	–

*Note*: * = *p* < 0.05; ** = *p* < 0.01; *** = *p* < 0.001.

**Table 2:** Mediation path analysis examining if confidence to intervene and educate in cases of animal abuse mediates the relationship between professional characteristics and the intent to intervene in cases of animal abuse (*n* = 145).

	*β* (STDYX)	SE	*p*-Value
Direct Effects			
Confidence to Intervene			
Time in Practice	−0.07	0.07	0.257
Perceived Seriousness of Animal Abuse	**0.17**	**0.08**	**0** **.031**
Research Knowledge	0.00	0.08	0.983
Legal Knowledge	0.00	0.09	0.975
Procedural Knowledge	**0.47**	**0.07**	**<** **0** **.001**
Number of Experiences Related to Animal Abuse	**0.21**	**0.08**	**0** **.006**
Intentions to Intervene			
Confidence to Intervene	**0.28**	**0.12**	**0** **.019**
Time in Practice	0.12	0.07	0.096
Perceived Seriousness of Animal Abuse	**0.45**	**0.09**	**0** **.000**
Research Knowledge	−0.03	0.08	0.678
Legal Knowledge	0.02	0.11	0.868
Procedural Knowledge	0.03	0.10	0.729
Number of Experiences Related to Animal Abuse	0.12	0.08	0.141
Indirect Effects			
Time in Practice—Confidence—Intentions to Intervene	−0.01	0.00	0.269
Perceived Seriousness—Confidence—Intentions to Intervene	0.06	0.04	0.091
Research Knowledge—Confidence—Intentions to Intervene	0.00	0.01	0.983
Legal Knowledge—Confidence—Intentions to Intervene	0.00	0.01	0.975
Procedural Knowledge—Confidence—Intentions to Intervene	0.09	0.03	0.033
Number of Experiences Related to Animal Abuse—Confidence—Intentions to Intervene	0.03	0.02	0.075

*Note*: SE = standard error; boldface indicates statistical significance.

## Data Availability

The data presented in this study are available on request from the corresponding author due to privacy concerns.
